# CRISPR-cas system: biological function in microbes and its use to treat antimicrobial resistant pathogens

**DOI:** 10.1186/s12941-019-0317-x

**Published:** 2019-07-05

**Authors:** Muhammad Abu Bakr Shabbir, Muhammad Zubair Shabbir, Qin Wu, Sammina Mahmood, Abdul Sajid, Muhammad Kashif Maan, Saeed Ahmed, Umer Naveed, Haihong Hao, Zonghui Yuan

**Affiliations:** 1 China MOA Laboratory for Risk Assessment of Quality and Safety of Livestock and Poultry Products, Huazhong Agricultural University, Wuhan, 430070 People’s Republic of China; 2 National Reference Laboratory of Veterinary Drug Residues and MOA Key Laboratory for the Detection of Veterinary Drug Residues in Foods, Huazhong Agricultural University, Wuhan, 430070 People’s Republic of China; 3 Quality Operation Laboratory at University of Veterinary and Animal Sciences, Lahore, 54600 Pakistan; 4 Department of Botany, University of Education, Bank Road Campus, Lahore, Pakistan; 5 College of Veterinary Sciences and Animal Husbandry, Abdul Wali Khan University, Mardan, 23200 Pakistan; 6 The Roslin Institute, University of Edinburgh, Edinburgh, Scotland UK

**Keywords:** CRISPR-cas system, Antimicrobial resistance, Cas9, Antibacterials, Intracellular infection

## Abstract

The development of antibiotic resistance in bacteria is a major public health threat. Infection rates of resistant pathogens continue to rise against nearly all antimicrobials, which has led to development of different strategies to combat the antimicrobial resistance. In this review, we discuss how the newly popular CRISPR-cas system has been applied to combat antibiotic resistance in both extracellular and intracellular pathogens. We also review a recently developed method in which nano-size CRISPR complex was used without any phage to target the *mecA* gene. However, there is still challenge to practice these methods in field against emerging antimicrobial resistant pathogens.

## Introduction

Antibiotic compounds have significantly impacted on modern medicine since their first introduction approximately 90 years ago. While antibiotics can overcome previously fatal infections, their irrational use in veterinary and agricultural fields poses a major threat as it results in tremendous flow of antibiotics into the environment [1]. This exposure of many antibiotics leads to enormous selective pressures that drive the spread and evolution of antimicrobial resistance genes in pathogenic and commensal bacteria [2, 3]. These antibiotic resistant genes enable bacteria to overcome antibiotics via different mechanisms, including the use of efflux pump, antibiotic molecule deactivation by enzymes, and chemical modification (ribosome and cell wall) to protect the cellular targets of antibiotics [4]. Taken together, these resistance mechanisms pose a threat to the efficacy of antibiotics used therapeutically [3]. The Center for Disease Control and Prevention reported in 2013 that antimicrobial resistant (AMR) pathogens infect more than 2 million people each year, resulting in 23,000 deaths [5]. It is also predicted that drug resistant pathogens will cause 10 million fatalities per year by the year 2050. This means drug resistant pathogens will cause more deaths than traffic accidents, diabetes and cancer [6]. A contributing factor to the development of antibiotic resistance is the ability of bacteria to adopt incredible phenotypic and genotypic heterogeneity for survival in adverse environments [6].

The main factor for AMR is the decline in novel antibiotic production: no new antibiotic class has been approved for Gram-negative bacterial infections in more than 45 years, and only 37 antibiotic drugs are currently in phase II or III clinical trials [7]. Further, antibiotic development, screening and testing is expensive and intensive resources are required [8]. These factors forced our hands to search for alternative treatments for AMR pathogens, including the development of a unique antibacterial arsenal with precise target capabilities. To achieve this, researchers have developed novel peptide and nucleic acid based antibacterials, bacteriophage therapies, bacteriocins, antibodies and anti-virulence compounds, among others [7]. In our review, we discuss the adaptive immune system of bacteria. Clustered regularly interspaced short palindromic repeats (CRISPR) system, and its role to overcome the growing AMR threat.

## Three types of CRISPR-cas system

The story of CRISPR-cas system began in 1987 when Nakata and colleagues reported a set of 29 nucleotide (nt) repeats in *E. coli* during their study of the *iap* gene [9]. By sequencing numerous microbial genomes in the next decade, additional repeat elements from the genomes of different archaeal and bacterial strains were also reported. Later, this unique family of inter-spaced repeat sequences were termed as clustered repeat elements [10]. In 2002, the term CRISPR was used by Mojica and Jansen [11]. A major breakthrough was in 2005, when spacer sequences were separated from direct repeats suggesting their phage association or extrachromosomal origins [12, 13]. In 2010, the basic function and mechanism of CRISPR-cas system has become clear. This system is comprised of a genetic locus with non-repetitive, spacer sequences and adjacent 6–20 genes that encode CRISPR-associated (cas) proteins [14, 15]. A number of researchers have begun to use the CRISPR-cas system for biotechnological applications and the generation of phage resistant dairy cultures [11].

**Fig. 1:**
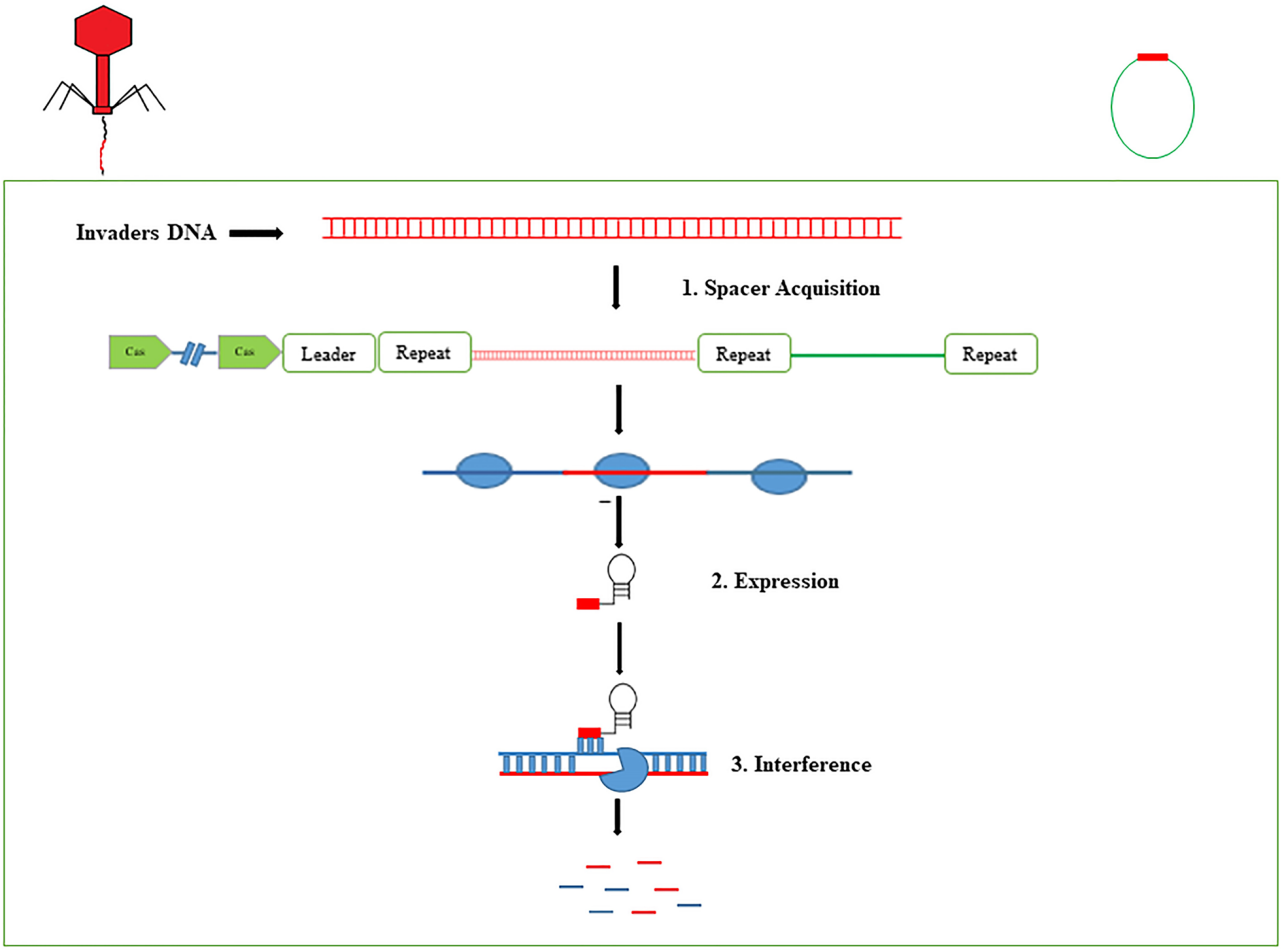
Mechanism of CRISPR-cas immunity divided into three stages. Stage 1: Spacer acquisition. In the first stage specific fragments of virus or plasmid double stranded are integrated at the leader end of CRISPR array on host DNA. A CRISPR array consists of unique spacer (red box) interspaced between repeats (blue box). Spacer acquisition occurs in the presence of cas1 and cas2 proteins, which are present near the vicinity of CRISPR array. Stage 2: Biogenesis of crRNA. In this stage RNA polymerase at leader end helps in the transcription of Pre-CRISPR RNA (Pre-crRNA) to mature crRNA. Stage 3: Interference. In the final stage, specific match between crRNA spacer and target sequence leads to the cleavage of foreign genetic elements (blue and red strips) [14, 15]

The CRISPR-cas system is an adaptive immune system of bacteria and archaea, which protects the bacteria from invaders, including bacteriophages or phages and mobile genetic elements (MGEs) [16]. The CRISPR-cas system degrades foreign genetic elements in three steps (Fig. 1). Adaptation or spacer acquisition [17] is the first stage in which spacer sequence after recognition is integrated into the CRISPR array. The second stage is biogenesis or expression of CRISPR RNA (crRNA), in which pre-CRISPR RNA (pre-crRNA) are transcribed by RNA polymerase (RNAP). These pre-crRNA are then cleaved into the small crRNA by specific endoribonucleases. Based on the crRNAs function, these are also known as guide RNAs [18, 19]. Interference [20] is the final stage in which crRNAs recognize and form base pair specific to foreign RNA or DNA with almost perfect complementarity [14, 15]. This leads to the cleavage of the crRNA-foreign nucleic acid complex. Conversely, if there is any mutation in proto-spacer adjacent motif (PAM) or mismatch between spacer and invader’s DNA, the cleavage does not occur and the host is susceptible to infection [21].

The CRISPR-cas system is divided into three subtypes: CRISPR-cas system type I, II and III. This classification was made on the basis of signature genes present in each type. For example, type I has cas3, type II has cas9 and type III has cas10 gene. However, it is important to note that all types and subtypes of CRISPR system have cas1 and cas2 proteins, since these two proteins play a key role in spacer [14, 15]. CRISPR-cas system types present in different bacterial species of interest are provided in Table 1.

**Table 1:** Presence of different CRISPR-cas system types in bacterial species

CRISPR-cas system	Bacterial species	References
Type I	*E. coli, P. aeruginosa, M. xanthus, B. halodurans, C. concisus, C. curves, C. fetus, C. hominis, C. rectus, Y. pestis, Salmonella specie, E. amylovora, P. acnes*	[18, 22–29]
Type II	*S. thermophilus, S. mutants, N. meningitides, C. jejuni, L. pneumophila, L. monocytogenes, F. novicida, S. pyogenes, S. aureus, M. gallisepticum, E. faecalis*	[21, 30–38]
Type III	*P. furiosus, S. epidermidis, M. tuberculosis*	[39–41]

## Type I system

CRISPR-cas type I system is present in most bacteria and archaea [25]. This system is further divided into six subtypes (A–F) encoding cas3 gene. Cas3 is a multi-domain protein with helicase and nuclease activity [42]. The Cas3 protein contains two domains: an N-terminal HD phophohydrolase for cleavage of DNA and a C-terminal DExH helicase domain to unwind double stranded DNA [14, 15]. These two domains work together to degrade invader DNA. However, cas3 alone is not able to identify invader’s DNA and protect cells from infection [18, 43]. In each subtype of the type I system, a number of specific subtype cas proteins assemble to form a complex known as crRNA guided surveillance complex or CRISPR associated complex for antiviral defense (CASCADE). These complexes play a role in the identification and binding of target sequence complementary to the crRNA spacer. The crRNA guided surveillance complex was first described in *E. coli* K12 (type I-E) [18, 43]. The complex is a combination of five cas proteins. Cas6e (previously named as Cse3 or CasE) helps in the maturation of crRNA. The mature crRNA remains attached to the CASCADE complex and has a role in the detection and cleavage of invader DNA [14, 15]. Similar type of complex was also reported in *S. solfataricus* [44]. Additionally, crRNA guided surveillance complexes have been identified in *Pseudomonas aeruginosa* (type I-F) [45] and *Bacillus halodurans* (type I-C) [46].

## Type II system

This system is present only in bacteria [25]. Compared to other CRISPR-cas types, the type II system is the simplest [21]. The CRISPR-cas type II system has four genes: cas1, cas2, cas9, and cas4 in the case of type II-B or csn2 in the case of type II-A. The characteristic protein of the type II system is cas9, which has a role in both crRNA biogenesis and cleavage of invader DNA [35]. The cas9 gene consists of two domains: RuvC and HNH domains [47]. The HNH domain helps in cleavage of DNA which is complement to crRNA guide, while the RuvC domain is involved in the cleavage of non-complement strand [47]. The biogenesis of crRNA in type II system requires a trans-activating crRNA (tracrRNA). The encoding of tracrRNA in *Streptococcus pyogenes* take place at the opposite strand of CRISPR-cas locus [14, 15]. Hybridization between crRNA repeats and tracRNA leads to the formation of double stranded RNA (dsRNA), which is identified and cleaved by cellular non-cas RNase III enzyme. The biogenesis of crRNA is inhibited by the deletion of cas9 gene; however, its role in crRNA biogenesis is unclear [35]. Jinek and colleagues demonstrated that cas9 enzyme requires both tracrRNA and crRNA for the cleavage of target DNA [47]. Notably, all domains necessary for DNA cleavage are fused in a single protein (cas9), which makes the CRISPR-cas type II system an ideal choice for genome manipulation [48].

## Type III system

Type III system has been divided into two subtypes: type III-A and type III-B [25]. This system is most commonly present in archaea, but it was also reported that the type III-B system is present only in conjunction with other CRISPR types. The CRISPR-cas type III system encodes both cas6 and cas10 genes. Cas10 is also known as repeat associated mysterious protein (RAMP), and is potentially involved in the maturation of crRNA and DNA cleavage [49]. Cas6 is an endoribonuclease which is not associated with CASCADE complex and works independently [50]. The CASCADE complex of type III system binds with mature crRNA and cleaves foreign RNA [51]. Furthermore, cas6 might be shared in those archaea which have type III system along with CRISPR-cas type I-A or I-B [52].

Although these two subtypes of type III CRISPR-cas system have similarities, it appears that these two systems target chemically different substrates. For example, type III-A system of *S. epidermidis* targets DNA while type III-B system present in *S. solfataricus* and *Pyrococcus furiosus* cleave RNA [14, 15]. This shows the diversity of CRISPR system present within the type III systems.

## Role of CRISPR-cas system in bacterial virulence

Several studies have shown that the CRISPR-cas system has additional functions apart from defending bacteria against invaders. This system also controls endogenous transcription and is involved in the regulation of bacterial pathogenicity. For example, *Francisella novicida*, a possible causative agent of disease in humans, replicates intracellularly by bypassing the host immune system. This bacterium has several mechanisms to subvert host macrophages and other immune cell functions. Upon the engulfment by macrophages, *F. novicida* enters the phagosome, a compartment having several antimicrobials and immune recognition receptors [53]. Toll-like receptor 2 (TLR2) is one of those receptors that can detect bacterial lipoproteins (BLPs) [34]. Activation of TLR2 initiates a pro-inflammatory response and recruits as well as activates immune cells, this ultimately helps in clearing the bacterial pathogen. *F. novicida* uses cas9, sacRNA (small, CRISPR associated RNA) and tracrRNA as regulators to repress the BLP expression [31]. Thus, this pathogen can survive within the host by preventing the activation of TLR2. However, *F. novicida* induces significant inflammatory response in the absence of these regulators, as it was reported that cas9, sacRNA and tracrRNA deletion mutants elicits a significant inflammatory response compared to wild type [31]. Additionally, it has also been reported that these mutants were not able to productively infect mice, further emphasizing the importance of CRISPR-cas system as a virulence regulator in *F. novicida* [31].

It was also reported that *Neisseria meningitides* uses cas9 for attachment to host cell surface and intracellular replication [31]. In addition, it has been reported that *Campylobacter jejuni* uses cas9 for attachment as well as for invasion in epithelial cells [32]. We hypothesize that the CRISPR-cas system not only facilitates *C. jejuni* attachment to host cells, but also protects this bacterium from the host’s innate complement system. Further, deletion of cas9 gene in *cst*-*II* positive *C. jejuni* results in almost complete loss of virulence [32]. The exact mechanism by which cas9 gene regulates virulence in these microorganisms is not yet known, but it is hypothesized that cas9 does not work alone to control virulence properties. Recently, a study reported that CRISPR-cas9 gene has a role in the regulation of several virulence associated genes and increases the virulence of *C. jejuni* [54].

## CRISPR-cas system involvement in antimicrobial resistance

There are several studies implicating the CRISPR-cas system in antimicrobial resistance. For example, this system promotes envelope integrity of *F. novicida* by the regulation of BLP. This leads to the development of resistance against several membrane stressors, including antibiotics [55]. A separate study found a relationship between competence systems (promotes gene acquisition) and CRISPR system: *Aggregatibacter actinomycetemcomitans* competent strains have CRISPR-cas systems, while non-competent bacterial strains lost their CRISPR immune system [56]. This finding revealed that the evolution of competence system and CRISPRs promotes genetic heterogeneity and the rise of new bacterial species. Similarly, it was suggested by Levin et al. [57] that bacteria having a CRISPR system might acquire resistance which could result in a population of bacteria with greater fitness than other variants.

It is also important to note that CRISPR system protects the host genome against invaders to maintain genetic homeostasis [58, 59]. Foreign genetic elements, such as plasmids and other conjugative elements, may carry beneficial genes that may increase bacterial fitness in the environment, such as virulence and antibiotic resistance.

Several studies have found a negative correlation between CRISPR-cas system and the presence of plasmids and phages, as explained in *Enterococcus*, *Campylobacter* and many group A *Streptococcus* species [60]. One study found that targeting of plasmid by CRISPR-cas system results in untoward effect in *S. epidermidis* regarding its antibiotic resistance [58, 61].

## Why CRISPR-cas system used to encounter antibiotic resistance threat?

Four major classes of DNA binding proteins have been engineered to achieve effective genome editing: meganucleases originated from microbial MGEs [62], transcription activator like effectors (TALEs) derived from bacteria (*Xanthomonas*) [63], Zinc finger nucleases (ZFNs) from eukaryotic transcription factors [11] and finally the RNA guided DNA endonucleases cas9 from CRISPR-cas type II system of bacteria [48].

Genome editing by meganucleases is not widely used due to low sequence specificity for target DNA [11]. ZFNs also have limitations, as they are difficult to design for binding to a desired sequence. Furthermore, ZFNs have limited target site selection. TALENs are easy to design due to their capacity to have longer DNA binding protein domains, allowing for high specificity of targeting. However, TALENs are much larger than ZFNs, and this size poses a complication for delivery into cells [64].

The Cas9 nuclease of the CRISPR-cas type II system uses a guide RNA to identify target DNA by Watson–Crick base pairing. Sequences present in CRISPR guide RNAs are specific to an invader sequence, meaning this sequence can be easily replaced by our desired sequence to retarget the CRISPR-cas9 nuclease [11].

Detailed study of the CRISPR-cas system has enabled researchers to insert, delete and mutate desired genes in virtually any species, and can even be used to correct genetic diseases in live animals [65]. Additionally, this system is now used in specific antibacterial preparations that can target AMR pathogens within complex populations of bacteria, allow antibacterial delivery to pathogenic bacteria, and in some cases deliver treatments to host cells infected with pathogenic bacteria. The CRISPR-cas system distinguishes between commensal and pathogenic bacterial species due to sequence specific targeting. The potential of CRISPR-cas system to counteract AMR pathogens is highlighted here.

## Irony: bacterial defense system against their own kind

The CRISPR-cas system can differentiate between commensal and pathogenic species due to highly specific sequence targeting. CRISPR guide RNAs can be designed to target virulence and chromosomal genes which are specific to pathogens, thereby enabling the CRISPR-cas system to be repurposed against bacteria instead of defending against invaders [66]. CRISPR-cas9 technology can be used to produce sequence specific antibiotics with the ability to target only AMR pathogens [67]. Cas9 is a double stranded DNA nuclease that can be programmed or used to cleave any DNA sequence [67]. Previously, scientists transformed *E. coli* and *Staphylococcus aureus* with a plasmid encoding cas9 guided RNAs that precisely degraded antibiotic resistant genes [47, 68]. Cas9 programmed with specific target sequences can enhance the cytotoxicity of resistant cells. This means AMR pathogens can be reverted to antibiotic sensitive cells by precise cleavage of resistant genes with the help of CRISPR-cas9 system.

The major obstacle in CRISPR-cas9 antibacterial delivery is delivering the complex (160-kDa protein-RNA) through the membrane of bacteria. In addition, how can this complex be delivered to both Gram-positive and Gram-negative bacteria? To solve this problem, many researchers used species-specific phages as vehicles for CRISPR-cas delivery. Phages are natural predators of bacteria that inject their DNA into bacteria. In 2014, it was reported that CRISPR-cas9 designed to target specific chromosomal genes of bacteria can be encapsulated into the capsids (protein coat) of inert phages by genetically encoding the phagemid. Phagemid is a plasmid designed to be packaged into the capsids of phage [69]. Another study reported that genetically modified phage having CRISPR-cas9 can target antibiotic resistance in *S. aureus* [70]. Taken together, these findings showed that CRISPR-cas9 antibacterials are highly specific for pathogenic bacteria and spare non-pathogenic species, which is a basic requirement for the development of new novel antibiotics. These in vitro findings highlight the attractiveness of phages as a means for CRISPR-cas9 delivery for the rapid killing of resistant pathogens.

Moreover, other groups have explored the potential of CRISPR-cas9 in removing resistant bacteria from complex bacterial populations [71]. Further work has engineered phage scaffolds to increase the host range expansion [72], and others have explored gene editing strategies to re-sensitize bacteria against antibiotics [65]. These results support the repurposing of CRISPR-cas9 machinery to be used against AMR infections and newly emerging bacterial strains. The CRISPR-cas9 is highly adaptable and programmable by altering the sequence of guide RNA. However, the methods used for encapsulation of both sgRNA and protein limit their practical use because of low loading and packaging efficiencies. Additionally, a requirement for high administration dosage may cause toxicity problems [73, 74].

To circumvent these problems, recent work has developed a method of non-viral genome editing, in which they successfully used nano-sized CRISPR complex to target the *mecA* gene. They used polymer derived cas9 protein which is a covalently modified with cationic polymer. They claimed that nano-sized CRISPR complex (Cr-Nanocomplex) were successfully formed without disturbing the CRISPR-cas9 activity of DNA cleavage. Cr-Nanocomplex specifically designed to target *mecA* gene. This gene is involved in methicillin resistance and can be delivered efficiently into the methicillin-resistant *S. aureus* (MRSA), and allow the efficient bacterial genome editing than the native cas9 complex or traditional lipid based formulation [75]. This work strongly suggest that CRISPR-cas9 can be repurposed to attack AMR pathogens.

However, these methods only address external and topically treated infections, like MRSA. Therefore, other strategies are needed for systemic intracellular pathogens to treat tissue and organ specific infections.

## Intracellular delivery of CRISPR-cas9 antibacterials

**Fig. 2:**
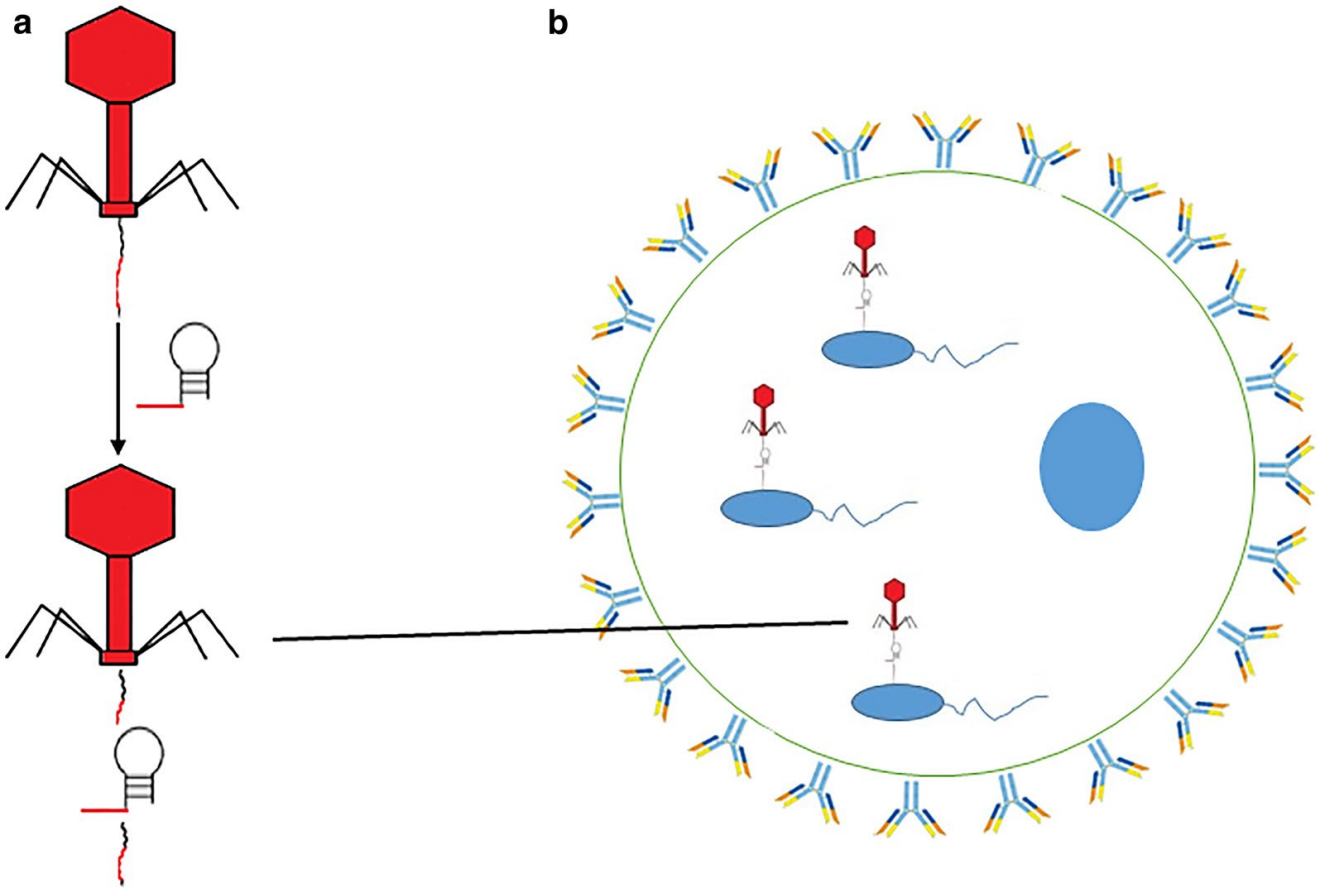
**a**, **b** CRISPR-cas9 antibacterials delivery to infected cell. **a** CRISPR-cas9 antibacterials encoded in bacteriophages. **b** CRISPR-cas9 encoded in phages were then introduced into the infected host cells to combat AMR pathogens

As previously explained, genetically encoded phage genomes can be used to deliver CRISPR-cas9 antibacterials into bacteria. When pathogenic bacteria are intracellular, the delivery of CRISPR-cas9 antibacterials becomes more challenging. In this scenario, CRISPR-cas9 encoded in phage must be used to specifically target infected cells, before its release into the bacteria residing within the cells. This delivery process is complicated by a specificity for both layers (host cell and intracellular bacteria) [8] as shown in Fig. 2.

After the development of new drug, its effectiveness to the matrices for efficient delivery and therapy are the greatest challenge. The modification of chemical properties of a drug for delivery reduces the treatment cost with some additional benefits, such as the bypassing of healthy cells and a lower dosage requirement. There are multiple approaches to use CRISPR-cas9 to edit the human host cell [76]. However, these strategies only deal with human target cells and do not address the challenges associated with delivery of CRISPR-cas9 encoded in phages to combat intracellular infections. Additionally, variation in size and structure of different phages needs to be considered. To overcome these challenges, CRISPR-cas9 offers a strategy that can help in the development of programmable antibacterials that can be modified genetically into a phage genome. Furthermore, delivery of CRISPR-cas9 into infected host cells by encoded phages provides an advancement over currently available delivery strategies.

It is known that phages have structural diversity; therefore, traditional strategies such as the use of nanoparticles as cargo are not practical. Different porous nanoparticles are used as cargo for drug delivery, but these methods are not effective when large and non-symmetrical phages are used due to limitations in pore size. To solve these issues, it is necessary to encapsulate bacteriophages to stabilize them for therapeutic purposes; previous work has shown it is possible to directly induce the self-assembly of phage encapsulation (bacterial cargoes) into lipid and silica based particle structures [77]. Chemical formulations to encapsulate, such as doping in silica and stabilizing proteins, can be modified by the use of different biological components. Silica based encapsulated phages can evade the immune system while retaining normal biological function. This strategy can help overcome the twofold barrier problem to treat intracellular bacterial infections such as *Burkholderia pseudomallei*.

CRISPR-cas9 encoding phages provide the opportunity for species-specific delivery of antibacterials. Furthermore, CRISPR-cas9 adaptability allows for rapid development of biologics to counteract AMR pathogen infections. It is now possible to build CRISPR-cas9 guide RNAs library to combat rapidly evolving AMR pathogens [8].

## Summary

AMR pathogens are a major public health concern worldwide. Different strategies have been developed to counter the rise of AMR. Among them, a newly developed technique known as CRISPR-cas system brings an arsenal in the warfare against resistant pathogens. With the help of CRISPR-cas system, scientists treat both extracellular (MRSA infection) and intracellular (*B. pseudomallei*) antibiotic resistant pathogens. However, it is still a challenge to apply CRISPR-cas9 antibacterials against non-laboratory resistant pathogens.

## Future perspective

1. Choosing a suitable combination of temperate and lytic phages being specific for sensitized pathogens is a big challenge. Thus there is a dire need to develop a universal means for efficient delivery of DNA into all pathogens.
2. Phages encoded with CRISPR-cas9 should also be used in non-laboratory strains without disrupting the native healthy microbiomes.

## Data Availability

All data is available in the manuscript.
